# RNA of *Enterococcus faecalis* Strain EC-12 Is a Major Component Inducing Interleukin-12 Production from Human Monocytic Cells

**DOI:** 10.1371/journal.pone.0129806

**Published:** 2015-06-17

**Authors:** Ryoichiro Nishibayashi, Ryo Inoue, Yuri Harada, Takumi Watanabe, Yuko Makioka, Kazunari Ushida

**Affiliations:** 1 Laboratory of Animal Science, Kyoto Prefectural University, Kyoto, Kyoto, Japan; 2 Department of Functional Foods, Combi Corporation, Saitama, Saitama, Japan; Indian Institute of Science, INDIA

## Abstract

Interleukin-12 (IL-12) is an important cytokine for the immunomodulatory effects of lactic acid bacteria (LAB). Using murine immune cells, we previously reported that the RNA of *Enterococcus faecalis* EC-12, a LAB strain exerting probiotic-like beneficial effects, is the major IL-12-inducing immunogenic component. However, it was recently revealed that bacterial RNA can be a ligand for Toll-like receptor (TLR) 13, which is only expressed in mice. Because TLR13 is not expressed in humans, the immuno-stimulatory and -modulatory effects of LAB RNA in human cells should be augmented excluding TLR13 contribution. In experiment 1 of this study, the role of LAB RNA in IL-12 induction in human immune cells was studied using three LAB strains, *E*.*faecalis* EC-12, *Lactobacillus gasseri* JCM5344, and *Bifidobacterium breve* JCM1192. RNase A treatment of heat-killed LAB significantly decreased the IL-12 production of human peripheral blood mononuclear cells on stimulation, while RNase III treatment revealed virtually no effects. Further, IL-12 production against heat-killed *E*. *faecalis* EC-12 was abolished by depleting monocytes. These results demonstrated that single stranded RNA (ssRNA) of LAB is a strong inducer of IL-12 production from human monocytes. In experiment 2, major receptor for ssRNA of *E*. *faecalis* EC-12 was identified using THP-1 cells, a human monocytic cell line. The type of RNA molecules of *E*. *faecalis* EC-12 responsible for IL-12 induction was also identified. IL-12 production induced by the total RNA of *E*. *faecalis* EC-12 was significantly reduced by the treatment of siRNA for TLR8 but not for TLR7. Furthermore, both 23S and 16S rRNA, but not mRNA, of *E*. *faecalis* EC-12 markedly induced IL-12 production from THP-1 cells. These results suggested that the recognition of ssRNA of *E*. *faecalis* EC-12 was mediated by TLR8 and that rRNA was the RNA molecule that exhibited IL-12-inducing ability in human cells.

## Introduction

Various effects of lactic acid bacteria (LAB) that are beneficial to health, such as antitumor [1, 2], anti-allergy [3, 4], and anti-infectious effects [5, 6], have been demonstrated in both human and animal models. Accordingly, LAB are frequently used as probiotics, live microorganisms, which confer health benefits to the host when administered in adequate amounts [7].

Recent evidence from in vivo studies has suggested that not only live but also heat-killed LAB exert many of the above-mentioned beneficial effects [8–12]. It is suggested that the effects of heat-killed LAB are derived from immunomodulation rather than alteration of the intestinal microbiota. Immunomodulatory effects originate from the recognition of LAB by immune cells and a resultant cytokine induction. Therefore, many studies have focused on the immunogenic components of LAB using both mouse and human immune cells [13–16].

Interleukin-12 (IL-12) has been suggested to be a critically important cytokine for immunomodulatory effects of LAB [17–19]. IL-12 is a proinflammatory cytokine produced by dendritic cells (DC), macrophages, and B cells in response to microbial stimuli. IL-12 stimulates interferon (IFN)-γ production in T cells and enhances the development of naive CD4^+^ T cells into Th1 cells [20]. Accordingly, IL-12 production is considered a good indicator of Th1-associated immunomodulatory effects such as antitumor, anti-allergy, and anti-infection.

Using murine immune cells, we have previously reported that the major IL-12p40-inducing immunogenic component of heat-killed *E*. *faecalis* EC-12 (EC-12) is its nucleic acids, particularly RNA [14]. Using *Staphylococcus aureus*, Oldenburg *et al*. [21] have reported that Toll-like receptor (TLR) 13, a TLR expressed in mice but not in humans, recognized a highly conserved bacterial 23S rRNA sequence “CGGAAAGACC” (nt 2055–2064 based on the *Escherichia coli* sequence). Many LAB, such as enterococci and lactobacilli, also have this sequence. Li *et al*. [22] have reported that RNA from *Lactobacillus salivarius* is recognized by TLR13. Because TLR13 is not expressed in humans, the immuno-stimulatory or-modulatory effects of LAB RNA in human cells should be augmented by excluding TLR13 contribution. Accordingly, the aim of this study is to clarify the role of LAB RNA in IL-12 induction in human immune cells.

In experiment 1, we evaluated the role of the cell wall, DNA, and RNA of three LAB strains, *E*.*faecalis* EC-12, *Lactobacillus gasseri* JCM5344, and *Bifidobacterium breve* JCM1192, in inducing IL-12 production from human immune cells. Single-stranded (ss) and double-stranded (ds) RNA were separately evaluated because dsRNA was suggested to be an immunogenic component of LAB [16]. Experiment 1 demonstrated that the ssRNA of LAB was a strong inducer of IL-12 production from human monocytes. In experiment 2, the major receptor that recognizes ssRNA from EC-12 was identified using THP-1 cells, a human monocytic cell line. The types of RNA molecules responsible for IL-12 induction were also identified. This study clearly demonstrated that ssRNA of EC-12 and at least two other LAB strains evaluated was an important immunogenic component for IL-12 production from human immune cells. The recognition of ssRNA of EC-12 was clearly mediated by TLR8, and rRNA, particularly 23S rRNA of EC-12 was the type of RNA that exhibited IL-12-inducing ability in human cells.

## Materials and Methods

### Experiment 1

#### Ethics statement

The research protocol for the present study was approved by the Ethical Committee of Combi Corporation (Saitama, Japan; Approval number: FF-001). Donors for peripheral blood collections have given written informed consent for their blood to be used for research purposes.

#### Peripheral blood mononuclear cell isolation and pre-culture

Peripheral blood was drawn from three healthy Japanese adults, collected in Venoject II vacuum blood collection tubes (heparinized blood collection tubes) (Terumo, Tokyo, Japan), and transferred to 50 mL centrifuge tubes in the presence of 2 mM ethylenediaminetetraacetic acid (EDTA). After two-fold dilution with phosphate buffered saline (PBS), peripheral blood mononuclear cells (PBMCs) were isolated by density gradient centrifugation on Percoll (d = 1.077) (GE Healthcare, Tokyo, Japan), at 500–800 g for 30 min. The remaining red blood cells were further lysed by ACK lysing solution (0.15 M NH_4_Cl, 10 mM KHCO_3_, and 0.1 mM Na_2_EDTA at pH 7.4). If required, the debris not dispersed after suspension of the cell pellet with PBS was carefully removed using a pipette. PBMCs were washed with PBS and suspended in culture medium [RPMI1640 (Nacalai Tesque, Kyoto, Japan) with 10% fetal calf serum (Equitech-Bio Inc., Kerrville, TX, USA), 100 U/mL penicillin and 100 μg/mL streptomycin (Nacalai Tesque)], at a density of 1 × 10^6^ cells/mL. One hundred microliters of PBMCs were seeded on each well of a 96-well cell culture plate (TPP, Trasadingen, Switzerland), and precultured in a humidified 5% CO_2_ incubator at 37°C for 4 h.

#### Monocyte isolation and pre-culture

Whole blood from one donor was obtained again and PBMC isolation was performed as described above. PBMCs were further cleaned by passing them through a 32-μm nylon mesh (Tokyo Screen, Tokyo, Japan) prior to monocyte isolation. Isolation of monocytes and monocyte-depleted PBMCs were performed by MACS technology using Monocyte isolation Kit II and a MS Column (Miltenyl Biotec, Tokyo, Japan), according to the manufacturer’s instructions. The monocytes and monocyte-depleted PBMCs were washed with PBS and suspended in a culture medium at a density of 2 × 10^5^ and 8 × 10^5^ cells/mL, respectively. One hundred microliters of monocytes in the culture medium were seeded on each well of a 96-well cell culture plate, and precultured in a humidified 5% CO_2_ incubator at 37°C for 4 h. Monocyte-depleted PBMCs were cultured in the same manner. Purities of both monocytes and monocyte-depleted PBMCs were greater than 85%, as assessed by staining with anti-CD14-PE [Clone: Tük4 (Acris Antibodies, Herford, Germany)].

#### Preparation of heat-killed bacteria and nuclease treatment

*L*. *gasseri* JCM5344 and *B*. *breve* JCM1192 were obtained from the Japan Collection of Microorganisms. *E*. *faecalis* EC-12 (International Patent Organism Depositary in Japan number, FERM BP-10284; GenBank Accession number, AB154827) was stored at Combi Corporation. All LAB were grown aerobically overnight at 37°C in MRS broth (Difco, Detroit, MI, USA), and washed with PBS, followed by centrifugation at 13,000 g for 3 min. Bacterial suspension in nuclease-free water (60 mg/mL wet weight) was heated at 98°C for 30 min using a thermal cycler (iCycler; Bio-Rad, Tokyo, Japan).

RNase A digests only ssRNA at high salt concentrations (higher than 300 mM NaCl); however, it digests ssRNA and dsRNA as well as RNA-DNA hybrids at low salt concentrations (0–100 mM NaCl) [23]. Therefore, RNase A treatment was performed at two different salt concentrations. Forty milligrams of heat-killed LAB were suspended either in 1 mL of low salt buffer (50 mM NaCl, 20 mM MnCl_2_, 50 mM Tris-HCl, and 1 mM dithiothreitol) or 1 mL of high salt buffer (300 mM NaCl, 20 mM MnCl_2_, 50 mM Tris-HCl, and 1 mM dithiothreitol), and RNase A (Invitrogen, Tokyo, Japan) was added at a final concentration of 200 μg/mL.

RNase III (final concentration 100 U/mL; New England Biolabs, Tokyo, Japan) that digests dsRNA was added to a high salt buffer containing 40 mg/mL of heat-killed LAB.

RNase-free recombinant DNase I (Roche, Tokyo, Japan) was used for DNase treatment. DNase I (final concentration: 200 U/mL) was added to the supplied reaction buffer containing 40 mg/mL of heat-killed LAB.

After 60 min of incubation at 37°C, nuclease-treated bacteria were washed with PBS, and resuspended in the culture medium.

#### Bacterial stimulation

Eight micrograms of LAB, with or without nuclease treatment, suspended in 100 μL of culture medium was added to each well of the 96-well cell culture plate in which PBMCs had been seeded.

To investigate endosomal involvement, PBMCs were treated with chloroquine (Invivogen, San Diego, CA, USA), which blocks endolysosomal nucleic acid recognition [24], at the final concentration of 10 μM. After a 30-min incubation, LAB without nuclease treatment was added as described above.

Cells were cultured with bacteria for 24 h in a humidified 5% CO_2_ incubator at 37°C. After incubation, the culture supernatants of PBMCs were collected and the concentration of IL-12 was measured using BD OptEIA Human IL-12 (p70) ELISA Set (BD Biosciences, San Diego, CA, USA).

Monocytes and monocyte-depleted PBMCs were stimulated using EC-12 and RNase A-treated EC-12 (in high salt buffer) because only a restricted number of purified monocytes was obtained. Eight micrograms of EC-12, with or without RNase A treatment, suspended in 35 μL of culture medium was added to each well of the 96-well cell culture plate in which MACS-isolated monocytes/monocyte-depleted PBMCs had been seeded. After 24 h of incubation, IL-12 concentration in the culture supernatant was measured as described above.

### Experiment 2

#### Total RNA preparation of EC-12

EC-12 was cultured and washed as described in experiment 1. Twenty milligrams of EC-12 (wet weight) was used for RNA extraction. Total RNA was extracted using RNeasy mini kit (Qiagen, Tokyo, Japan). Procedures, including on-column DNase digestion with the RNase-free DNase set (Qiagen), were performed according to the manufacturer’s instruction, with minor modifications; disruption of the bacteria was performed with 50–100 mg of zirconia beads (500 μm in diameter; BioSpec Products, Bartlesville, OK, USA) by Micro Smash MS-100 (5,000 rpm for 30 sec × 2; Tomy seiko, Tokyo, Japan).

Total RNA from EC-12 was fragmented to <150 nucleotides by using Magnesium RNA Fragmentation Module (New England Biolabs) because all reported endosomal receptors for ssRNA (TLR7 and TLR8) appeared to recognize 10–30 nucleotide sequences [25, 26] and unpredictable secondary or tertiary structure would be formed in case long nucleotides. Thirty micrograms of RNA, in 100 μL of the supplied RNA fragmentation buffer, was incubated for 25 min at 98°C. To stop the reaction, 1/10^th^ volume 10× RNA Fragmentation Stop Solution was added on ice.

Purification of the fragmented RNA was performed by ethanol precipitation. One-tenth volume of 3 M sodium acetate, 1/50^th^ volume of 5 mg/mL glycogen solution, and 3 volumes of 100% ethanol were added and incubated for at least 30 min at −20°C. The mixture was centrifuged at 20,400 g for 40 min at 4°C and the supernatant was carefully removed. The precipitate was washed with 70% ethanol and suspended in nuclease-free water. The size of fragmented RNA was confirmed using denaturing polyacrylamide gel electrophoresis (12% polyacrylamide, 8 M urea, and 1× Tris-acetate-EDTA buffer).

#### Maintenance and differentiation of THP-1 cells

Human monocytic cell line THP-1 cells (no. 88081201, European Collection of Cell Cultures, Salisbury, UK) were maintained in culture medium in a humidified 5% CO_2_ incubator at 37°C. To obtain a macrophage-like phenotype, THP-1 cells were differentiated by phorbol 12-myristate 13-acetate (PMA). Subconfluent THP-1 cells were suspended in the culture medium containing 10 ng/mL PMA (Sigma-Aldrich, Tokyo, Japan) and seeded on a 96-well cell culture plate at a density of 4.0 × 10^4^ cells/well.

#### RNA interference

After a 20-h incubation with PMA, the culture medium of THP-1 cells under differentiation was replaced with 100 μL of fresh culture medium, and RNA interference was performed.

Two TLR7 siRNA (Stealth siRNA, HSS121963 and HSS121964), two TLR8 siRNA (Stealth siRNA, HSS122021 and HSS122022), and a control siRNA (Stealth RNAi siRNA Negative Control, Med GC) were purchased from Invitrogen. First, 6 μL of Lipofectamine RNAiMAX (Invitrogen) was diluted in 100 μL of Opti-MEM (Invitrogen). Second, 4 μL of 20 μM siRNA stock solution was diluted in 100 μL of Opti-MEM and mixed with the abovementioned 100 μL of Opti-MEM containing RNAiMAX. After a 5-min incubation, 10 μL of RNAiMAX/siRNA mixture was added directly to the PMA-differentiated THP-1 cells. After 20 h of further incubation, the culture medium was replaced with 200 μL of fresh culture medium, supplemented with 35 ng/mL recombinant IL-6 (Tonbo Biosciences, San Diego, CA, USA) to obtain functional expression levels of TLR7 and TLR8 (S1 Fig) [27].

After 6 h of stimulation with IL-6, the culture medium of differentiated THP-1 cells was replaced with 100 μL of fresh culture medium and the total fragmented RNA from EC-12 was transfected as follows.

More than 45% knockdown of the target gene expression was confirmed prior to this experiment (P < 0.01; S1 Fig).

#### Transfection of total fragmented RNA

Polycationic polypeptide poly-L-arginine (pLa) was used to focus on endosomal receptors [28]. One microliter of pLa solution (4.5 mg/mL in PBS; P7762 from Sigma-Aldrich) was added to 110 μL of Opti-MEM, containing 3 μg of RNA, and incubated for 20 min. Thirty five microliters of the RNA/pLa mixture was added to each well of the 96-well cell culture plate, in which differentiated THP-1 cells had been seeded (final RNA concentration: 7 μg/mL).

Cells were cultured for 24 h in a humidified 5% CO_2_ incubator at 37°C. IL-12 concentration in the culture supernatant was measured as described in experiment 1.

#### Preparation of 23S, 16S rRNA, and mRNA from EC-12

Total RNA extraction of EC-12 was performed as described above. The total RNA obtained was separated using electrophoresis through 1.5% agarose gels (1× Tris-borate-EDTA buffer) and 23S rRNA and 16S rRNA were excised using a sterilized blade. Zymoclean Gel RNA Recovery Kit (Zymo Research, Orange, CA, USA) was used for the recovery/purification of 23S rRNA and 16S rRNA from the gel, according to the manufacturer’s instructions, with the following modification: Mini Plus Column (Viogene, Taipei, Taiwan) was used instead of the supplied Zymo-Spin IC Column to yield a higher RNA concentration. Fragmentation of RNA, purification of fragmented RNA, and assessment of size were performed as described above.

Purified mRNA was obtained from total RNA by sequential removal of rRNA and small RNA with the MICROBExpress Bacterial mRNA Purification Kit (Ambion, Tokyo, Japan) and the MEGAclear Kit (Ambion), according to the manufacturer’s instructions.

All RNA molecules were eluted in nuclease-free water.

#### Transfection of fragmented 23S rRNA, 16S rRNA, and mRNA

Because the length of 23S rRNA (2909 bases) is approximately two times as large as that of 16S rRNA (1522 bases), the concentrations of 23S rRNA and 16S rRNA were adjusted in molarity instead of mass concentration (w/v). Molecular weight of 23S rRNA and 16S rRNA were calculated by using the Endmemo software (http://endmemo.com/bio/dnacopynum.php).

After 40 h of incubation with PMA, the culture medium of differentiated THP-1 cells was replaced with 200 μL of fresh culture medium, supplemented with 35 ng/mL of recombinant IL-6. Following 6 h of stimulation at 37°C, the culture medium was replaced with 100 μL of fresh culture medium. Fifteen point seven microliters of pLa solution (4.5 mg/mL) was added to 1,730 μL of PBS, containing 50 pmol (47.2 μg) of 23S rRNA or 96 pmol (47.3 μg) of 16S rRNA. After 20 min of incubation, 17.5 μL of the 23S rRNA/pLa mixture was added to each well of the 96-well cell culture plate, in which differentiated THP-1 cells had been seeded (4 pmol/mL), while 9.1 or 17.5 μL of the 16S rRNA/pLa mixture was added (4 pmol/mL or 7.7 pmol/mL). The final media volume was adjusted to 125 μL.

As molarity of mRNA is hard to predict, mRNA was transfected at the same two mass concentrations with 16S rRNA (2 μg/mL and 3.8 μg/mL).

After 24 h of incubation, IL-12 concentration in the culture supernatant was measured in the same manner as described in experiment 1.

### Statistical analysis

Data are presented as mean values with standard deviations. Statistical analyses were performed using Statcel statistical software (OMS, Saitama, Japan). The effect of experimental treatments was analyzed using one-way ANOVA. When significant differences were detected, Scheffe's F-test post hoc comparison was used. P values of less than 0.01 were considered statistically significant.

## Results

### Experiment 1

#### Effect of nuclease treatment of LAB on IL-12-inducing ability in human PBMCs

**Fig 1:**
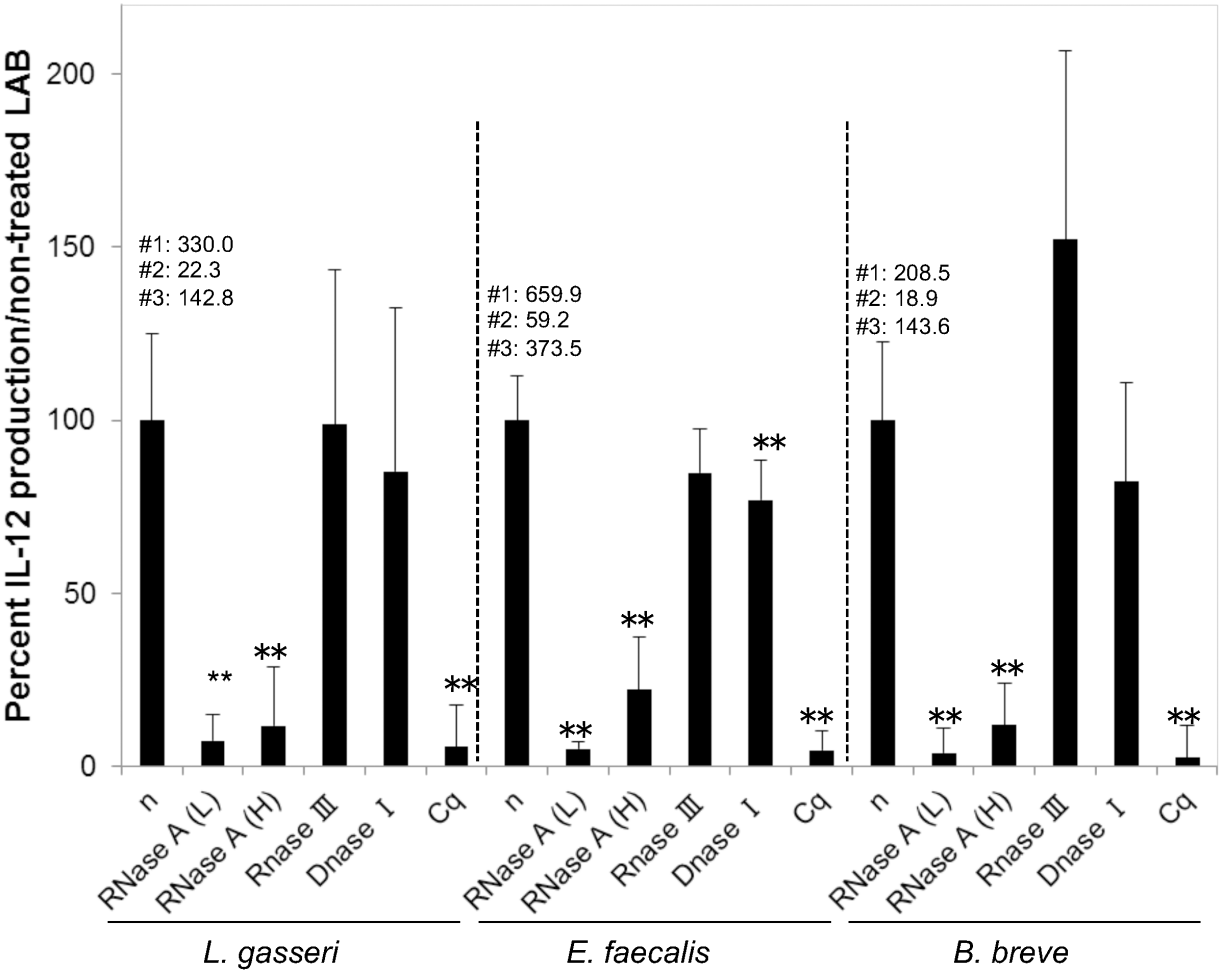
Effect of nuclease treatment of LAB on IL-12-inducing ability in human PBMCs. Heat-killed *L*. *gasseri* JCM5344, *E*. *faecalis* EC-12, and *B*. *breve* JCM1192 were treated with or without nuclease and co-cultured with PBMCs obtained from three donors, for 24 h. The IL-12 protein concentration in the culture supernatant was measured by enzyme-linked immunosorbent assay. Data are expressed as relative percentage to non-treated LAB. The value above the bars of non-treated LAB indicates the mean value of IL-12 concentration (pg/mL) in each donor. **n**: non-treated, **RNase A (L):** RNase A treatment in low salt buffer (digests all RNA), **RNase A (H):** RNase A treatment in high salt buffer (digests ssRNA), **Cq**: chloroquine treatment of PBMCs prior to bacterial stimulation. **: P < 0.01 vs non-treated LAB, mean ± SD, n = 3 per donor for each condition.

The effect of RNase A treatment was obvious irrespective of the LAB strains evaluated (Fig 1); RNase A treatment of LAB in both high and low salt buffers drastically decreased IL-12 production in human PBMCs on stimulation. Production of IL-12 induced by RNase A-treated LAB was lesser than 20% of that induced by non-treated LAB (P < 0.01). A slightly greater reduction in IL-12 production was observed in LAB treated with RNase A in low salt buffer than in those treated with RNase A in high salt buffer; however, no significant difference was detected between these two buffer salt concentrations. Treatment of LAB with RNase III showed no considerable effects on IL-12 production.

IL-12 induced by LAB treated using DNase I was approximately 80% of that induced by non-treated LAB. A significant reduction using DNase I treatment was observed in the case of EC-12 (P < 0.01).

Chloroquine completely blocked IL-12 production irrespective of the LAB evaluated strains (P < 0.01).

#### IL-12 production from human monocytes and monocyte-depleted PBMCs against EC-12

**Fig 2:**
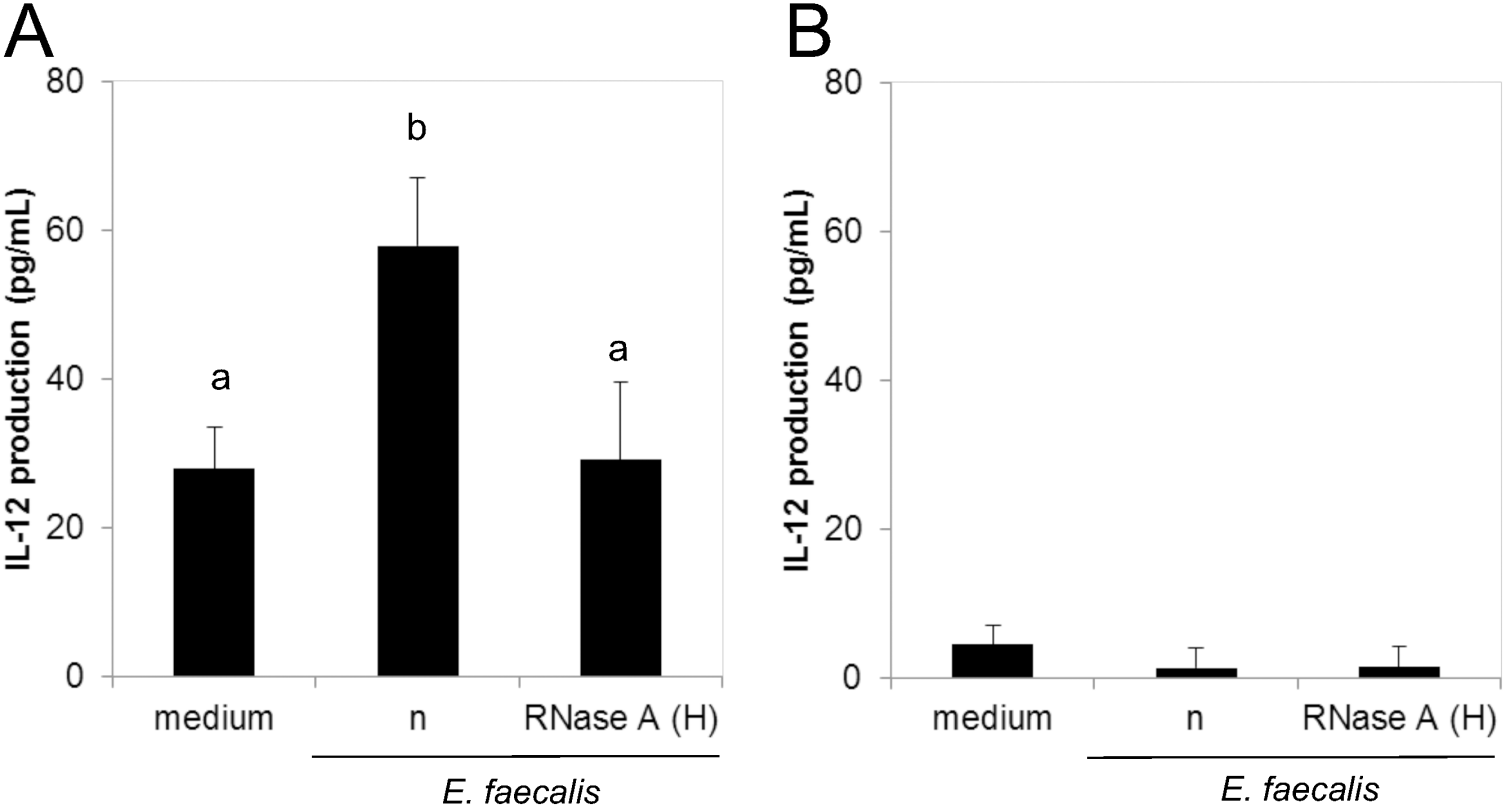
IL-12 production from human (A) monocytes and (B) monocyte-depleted PBMCs against *E*. *faecalis* EC-12. Heat-killed *E*. *faecalis* EC-12 was treated with or without RNase A in high salt buffer and co-cultured with **(A)** monocytes or **(B)** monocyte-depleted PBMCs for 24 h. The IL-12 protein concentration in the culture supernatant was measured by enzyme-linked immunosorbent assay. Bars sharing the same letter are not significantly different at P < 0.01, mean ± SD, n = 4.

EC-12 manifested the same IL-12-inducing ability in monocytes as that in PBMCs (P < 0.01). The IL-12 protein concentration in the culture supernatant of the monocytes co-cultured with EC-12 was 57.9 ± 9.1 pg/mL, which was approximately two times as large as that of the control cells (the non-stimulated intact monocytes). The IL-12-inducing ability of EC-12 was almost completely abolished by RNase A treatment in high salt buffer (29.1 ± 10.5 pg/mL) (Fig 2A).

On the other hand, virtually no IL-12 production was observed against EC-12 irrespective of nuclease treatment in the case of monocyte-depleted PBMCs (Fig 2B).

### Experiment 2

#### Identification of the major receptor responsible for recognition of EC-12 RNA

**Fig 3:**
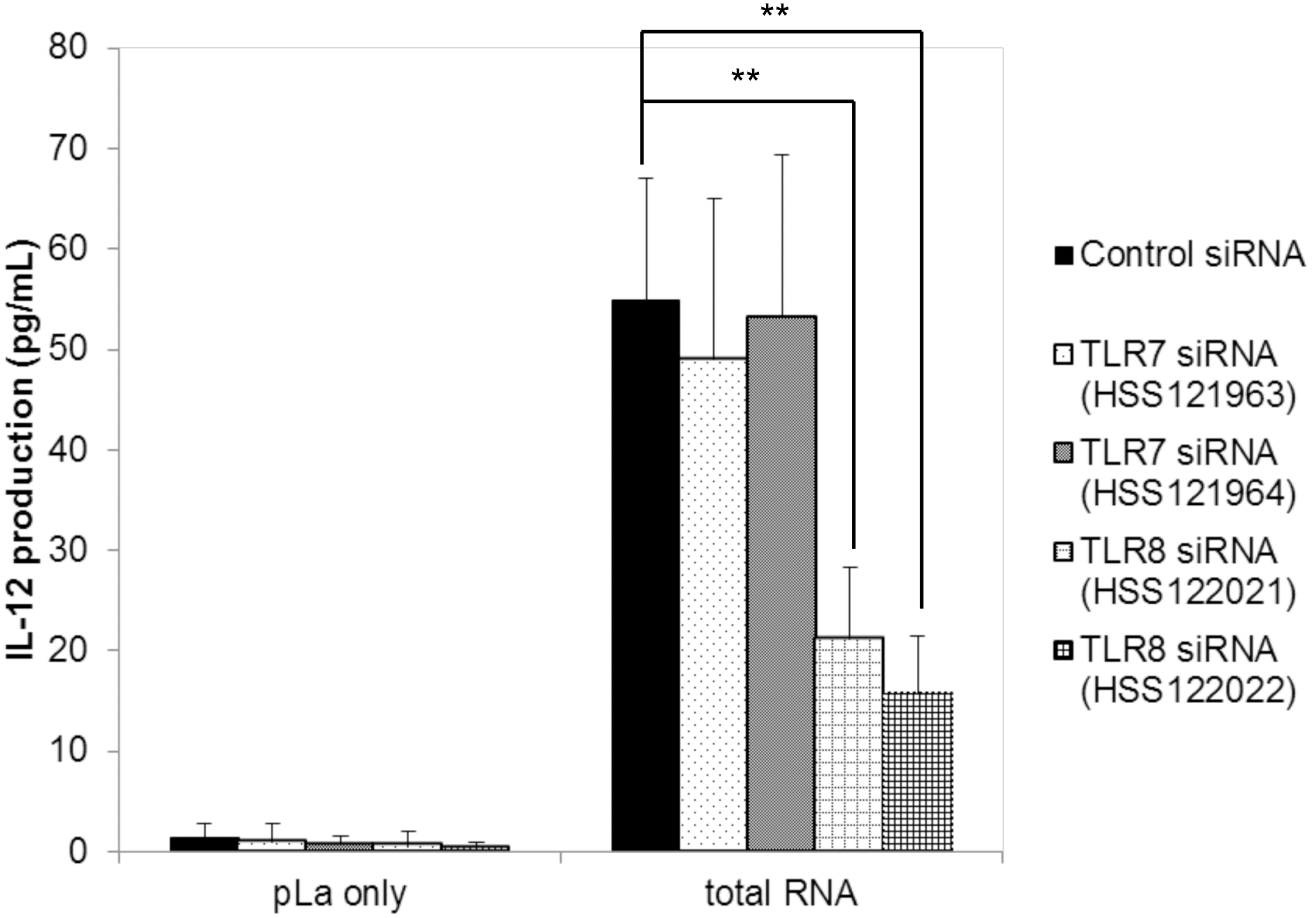
Effect of siRNA treatment on IL-12 production against total RNA of *E*.*faecalis* EC-12. siRNA treatment was performed as described in Material and Methods. Fragmented total RNA (7 μg/mL) from *E*. *faecalis* EC-12 was transfected using pLa to PMA-differentiated THP-1 cells after siRNA treatment. After 24 h of incubation, the concentration of IL-12 protein in the culture supernatant was measured using enzyme-linked immunosorbent assay. **: P < 0.01, mean ± SD, n = 6.

The production of IL-12 induced by the transfected total RNA of EC-12 was significantly reduced by the treatment of both siRNAs for TLR8 (P < 0.01); however, neither TLR7 siRNAs showed the same effect on IL-12 production. The IL-12 production induced by the total RNA of EC-12 was approximately 70% lesser in the TLR8 down-regulated cells than in the control cells (Fig 3).

#### Identification of the major RNA molecule inducing IL-12 production

**Fig 4:**
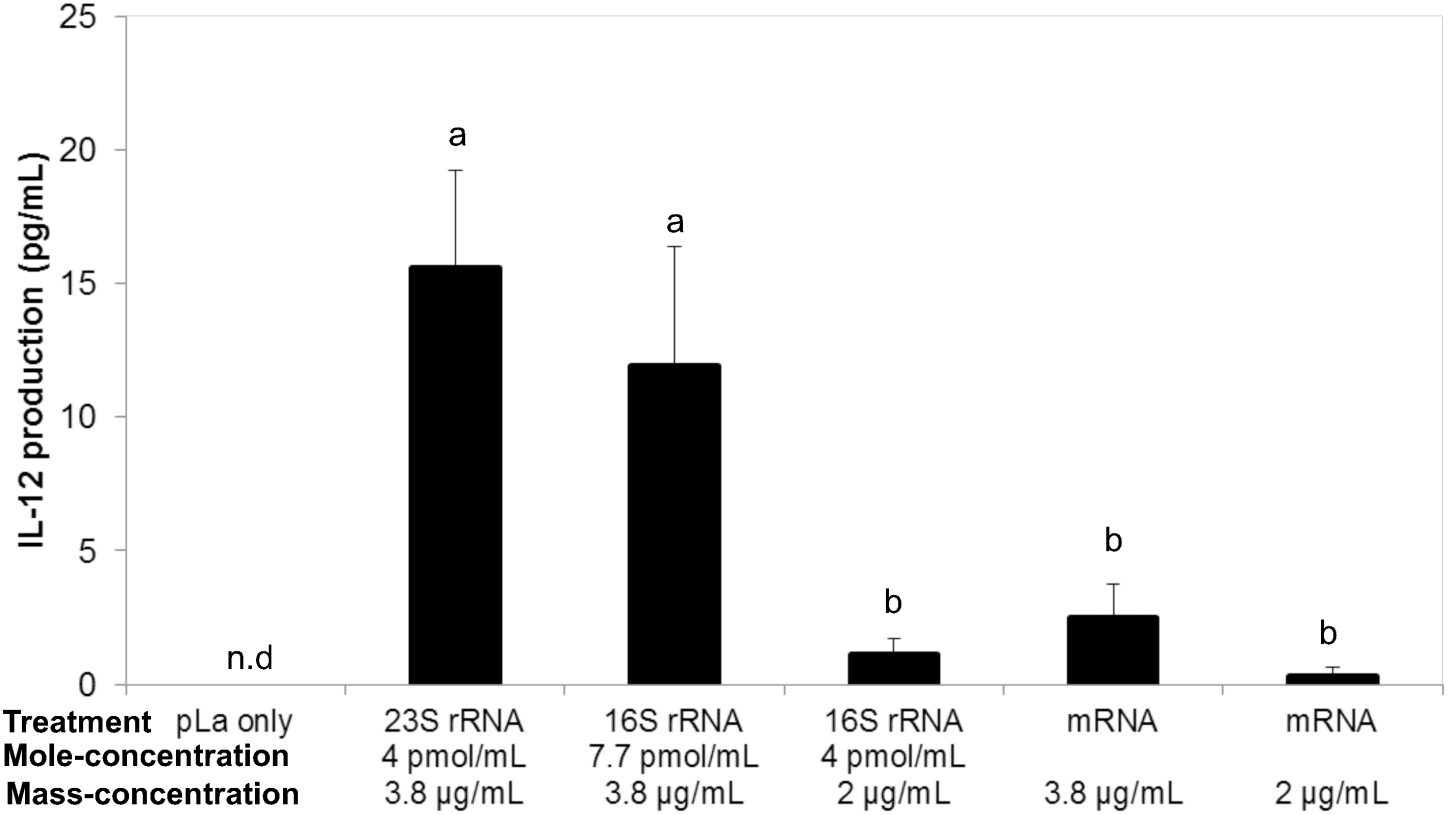
Effect of stimulation by rRNA and mRNA from *E*. *faecalis* EC-12 on IL-12 production. Fragmented 23S rRNA and 16S rRNA, and mRNA from *E*. *faecalis* EC-12 was transfected using pLa to PMA-differentiated THP-1 cells. After 24 h of incubation, the concentration of IL-12 protein in the culture supernatant was measured using enzyme-linked immunosorbent assay. Bars sharing the same letter are not significantly different at P < 0.01, mean ± SD, n = 4.

When cells were stimulated using 23S rRNA, significantly higher IL-12 production was observed than in the case of cells stimulated using equimolar 16S rRNA and equimass mRNA (P < 0.01). In addition, between equimass 23S rRNA and 16S rRNA, IL-12 concentration in the culture supernatant was still higher in 23S rRNA-stimulated cells than in 16S rRNA-stimulated cells (23S: 15.6 ± 3.6 pg/mL vs 16S: 11.9 ± 4.5 pg/mL; Fig 4).

## Discussion

IL-12 induction in immune cells is suggested to be an important initial event for LAB to exert immunomodulatory effects [17–19]. In this study, the role of LAB RNA in inducing IL-12 production was evaluated using human immune cells.

In experiment 1, the role of RNA from EC-12 and two other LAB strains in IL-12 induction in human PBMCs was evaluated by digesting bacterial RNA or DNA with nuclease. Because viable bacteria would not allow nucleic acid digestion by extracellularly-added nuclease, heat-killed LAB were used, as described in our previous study [14]. Although most of the RNA in the bacteria is single stranded, Kawashima *et al*. [16] have reported that some LAB induced IFN-β from mouse bone-marrow-derived DC in a TLR3-dependent manner (recognizing dsRNA). In addition, Hovden *et al*. [29] have also reported the dependency of penicillin-killed *Streptococcus pyogenes* (OK432) on the TLR3 pathway to induce IL-12 production from human monocyte-derived DC. Therefore, the roles of ssRNA and dsRNA were separately evaluated in this study.

Digestion of RNA in both high and low salt concentrations drastically reduced more than 80% of the IL-12-inducing ability in all LAB strains evaluated. Although RNase A treatment in low salt buffer showed a slightly higher reduction rate than in high salt buffer, no significant difference was detected between these two buffer salt concentrations. In addition, treatment of LAB with RNase III showed no significant effect on IL-12-inducing ability in human PBMCs. In case EC-12, the effect of nuclease treatments on IL-12-inducing ability was also confirmed by a flow cytometry analysis (S2 Fig). Therefore, the major RNA having IL-12-inducing ability is ssRNA and not dsRNA.

The reduction in IL-12 concentration achieved by DNase I treatment was much lower than that achieved by RNase A treatment (approximately 20%). Thus, for at least three of the LAB strains evaluated, ssRNA appeared to have almost unique immunogenic components to initiate IL-12 production from human PBMCs.

Human PBMCs consist of various cell types, including T cells, B cells, NK cells, plasmacytoid DC (pDC), myeloid DC (mDC), and monocytes. We attempted to identify the major cells in PBMCs that recognize EC-12 and subsequently produce IL-12. Ablasser *et al*. [28] have reported that IL-12 production from PBMCs using immunostimulatory oligo-RNA is highly dependent on monocytes. Therefore, we focused on monocytes in this study. MACS-isolated monocytes produced IL-12 in response to EC-12, and this response was abolished by RNase A treatment of EC-12, same as that observed in PBMCs. However, the stimulation of monocyte-depleted PBMCs by EC-12 induced no IL-12 production. We confirmed the same phenomenon in CD14^-^ cells isolated using a cell sorter (S3 Fig). In addition, flow cytometry analyses clearly showed that the cells producing IL-12 on stimulation were CD14^+^ cells (S2 Fig). These facts clearly indicate that in PBMCs, the ssRNA of EC-12 was almost solely recognized by monocytes.

Because chloroquine treatment of PBMCs completely abolished IL-12 production by all LAB strains evaluated, TLR7 and/or TLR8 expressed in endosomes [30] were considered as the most likely receptor-candidates for LAB ssRNA recognition. Indeed, recognition of viral ssRNA by human TLR8 has been demonstrated by Heil *et al*. [25]. The siRNA treatment in Experiment 2 clearly demonstrated that the recognition of total RNA from EC-12 largely depends on TLR8, but not on TLR7, in differentiated THP-1 cells, at least for the production of IL-12. There are only three previous reports that have shown recognition of bacterial RNA by human TLR7 [31, 32] and/or TLR8 [31, 33]. In addition, only two pathogenic bacterial species, *Borrelia burgdorferi* [32, 33] and *Escherichia coli* [31], have been examined, and IL-12 production was not evaluated in those reports. To the best of our knowledge, this study is the first to demonstrate the recognition of single stranded bacterial RNA by human TLR8 to induce IL-12 production from beneficial nonpathogenic bacteria.

The type of RNA molecules from bacteria, which induce cytokine production from human or mouse immune cells is controversial. Using human PBMCs, Eberle *et al*. [34] have reported that 16S rRNA and total RNA from *E*. *coli* have IFN-α-inducing potency. However, Li *et al*. [22] have reported that 23S rRNA from *L*. *salivarius* mainly induced IL-1β production from mouse macrophages. In addition, Mancuso *et al*. [35] have demonstrated that both mRNA and rRNA, particularly the former, from group B *Streptococcus*, are potent inducers of IFN-β in mouse conventional DC (cDC). Accordingly, the IL-12-inducing ability of 23S rRNA, 16S rRNA, and mRNA of EC-12 was evaluated in Experiment 2. It was obvious from our study that rRNA, but not mRNA, of EC-12 induced IL-12 production in differentiated THP-1 cells. In addition, 23S rRNA was apparently more potent in inducing IL-12 production from differentiated THP-1 cells than 16S rRNA.

In conclusion, this study demonstrates that ssRNA of EC-12 and at least two other LAB strains evaluated is an important immunogenic component for IL-12 production from human monocytic cells. The recognition of ssRNA of EC-12 was clearly mediated by TLR8 and rRNA; particularly, 23S rRNA of EC-12 was the RNA molecule that exhibited IL-12-inducing ability in human cells.

Further, ssRNA of EC-12 is the common immunogenic component that induces IL-12 production in both human and mouse immune cells, while its recognition is mediated by different TLRs: TLR8 in humans and TLR7 and TLR13 in mice [14, 22].

## Supporting Information

S1 Fig Real-time RT-PCR analysis of TLR7 and TLR8 expression following 6 h of IL-6 priming. siRNA treatment was performed as described in Materials and Methods. Total RNA was extracted and expression of TLR7 and TLR8 was analyzed by real-time RT-PCR. Expression level of β-actin was used as an internal control. Data are expressed as a percentage expression levels to the cells treated by control siRNA. Expression levels of non-IL-6 priming cells and cells treated with control siRNA, without IL-6 priming, are shown for convenience. **: P < 0.01, mean ± SD, n = 8.
(TIF)

S2 Fig Flow cytometry analysis on IL-12-producing cells in human PBMCs on stimulation by *E*. *faecalis* EC-12. Human PBMCs were stimulated by heat-killed *E*.*faecalis* EC-12 treated with or without nuclease (see legend of Fig 1) for 20 h in the presence of brefeldin A (10 μg/mL). PBMCs preparation and nuclease treatment of heat-killed EC-12 were performed as described in Materials and Methods. After the incubation, PBMCs were stained by anti-CD14-PE [Clone: Tük4 (Acris Antibodies)] followed by anti-IL-12p40-Alexa Fluor 647 [Clone:C11.5 (BioLegend, Tokyo, Japan)] according to the protocol provided by BioLegend (http://www.biolegend.com/media_assets/support_protocol/Intracellular_Staining_Protocol_041515.pdf). Analyses were performed on a BD Accuri C6 cytometer (BD Bioscience).
(TIF)

S3 Fig IL-12 production from human CD14^-^ cells against *E*. *faecalis* EC-12. Heat-killed *E*. *faecalis* EC-12 was co-cultured for 24 h with CD14^-^ cells sorted by a JSAN cell sorter (Bay Bioscience, Kobe, Japan). The IL-12 protein concentration in the culture supernatant was measured by enzyme-linked immunosorbent assay. mean ± SD, n = 4.
(TIF)
