# Supplementary figures and images for: RNA of Enterococcus faecalis Strain EC-12 Is a Major Component Inducing Interleukin-12 Production from Human Monocytic Cells
Source: PLoS One. 2015 Jun 17;10(6):e0129806. doi: 10.1371/journal.pone.0129806 (PMC4470910; doi:10.1371/journal.pone.0129806)

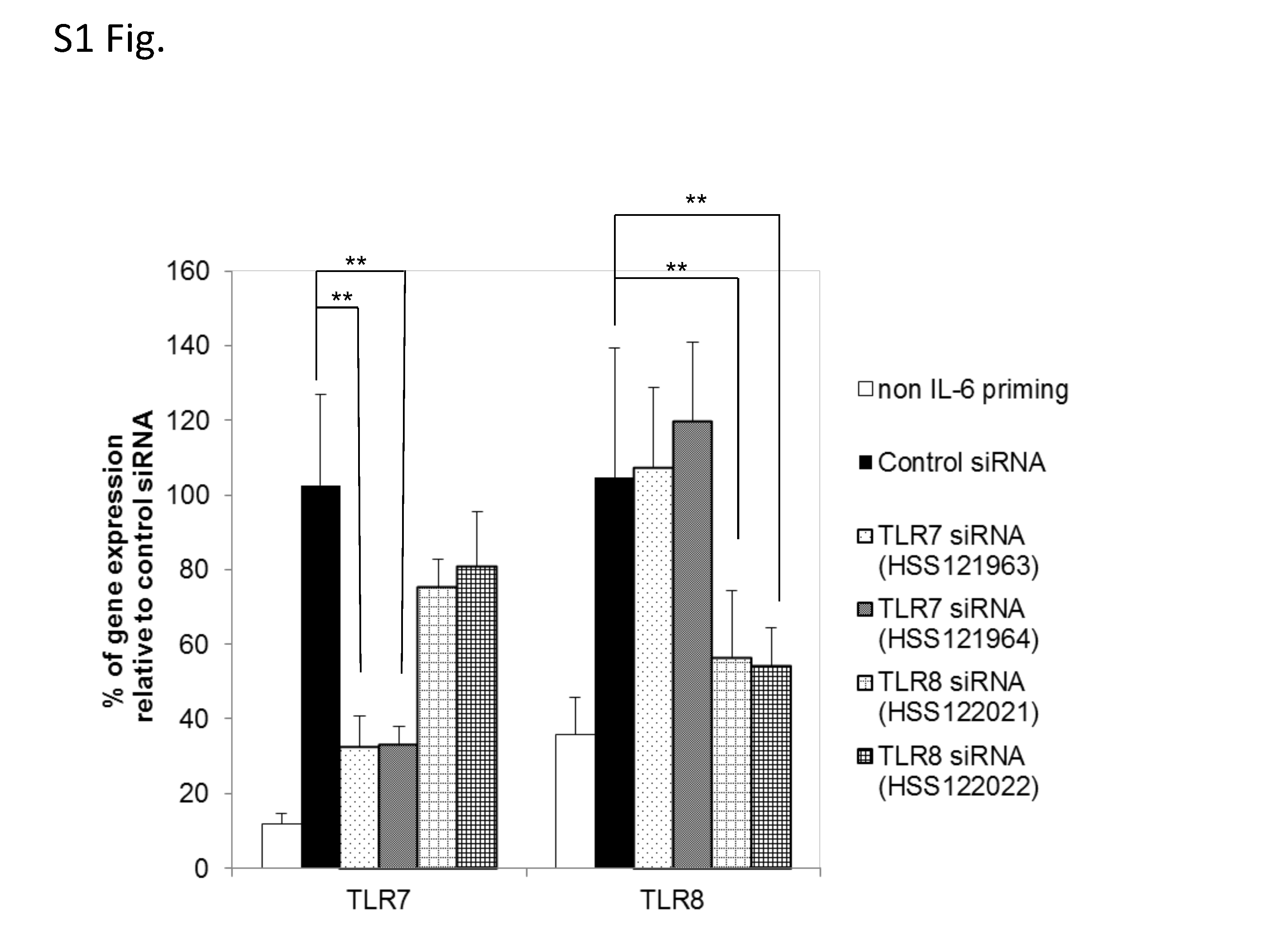

Supplement: S1 Fig — siRNA treatment was performed as described in Materials and Methods. Total RNA was extracted and expression of TLR7 and TLR8 was analyzed by real-time RT-PCR. Expression level of β-actin was used as an internal control. Data are expressed as a percentage expression levels to the cells treated by control siRNA. Expression levels of non-IL-6 priming cells and cells treated with control siRNA, without IL-6 priming, are shown for convenience. **: P < 0.01, mean ± SD, n = 8. (TIF) [file pone.0129806.s001.tif]

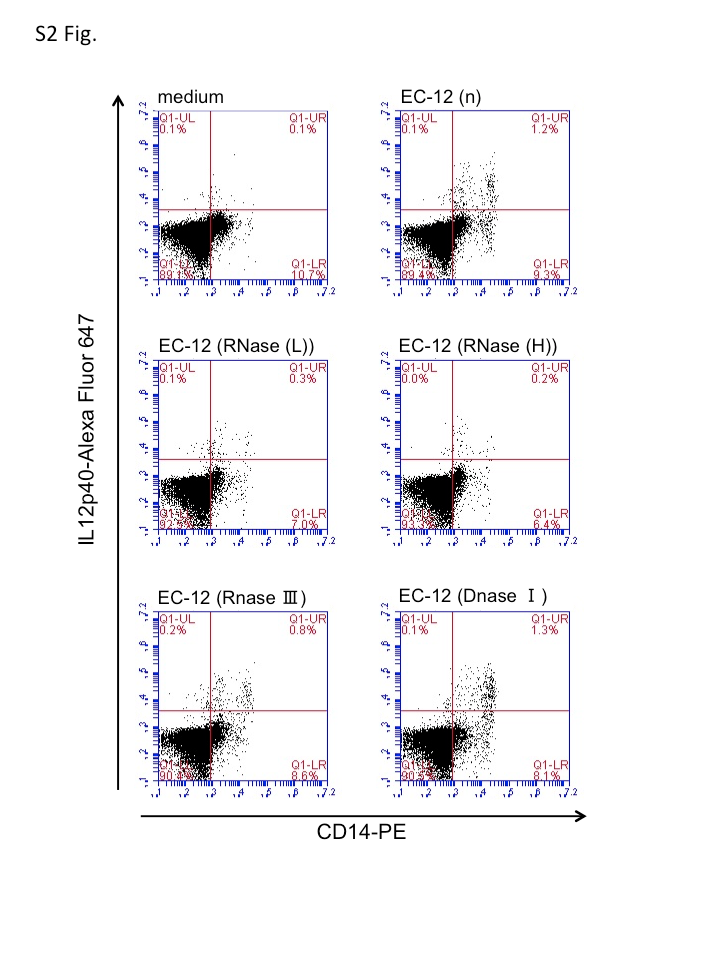

Supplement: S2 Fig — Human PBMCs were stimulated by heat-killed E.faecalis EC-12 treated with or without nuclease (see legend of Fig 1) for 20 h in the presence of brefeldin A (10 μg/mL). PBMCs preparation and nuclease treatment of heat-killed EC-12 were performed as described in Materials and Methods. After the incubation, PBMCs were stained by anti-CD14-PE [Clone: Tük4 (Acris Antibodies)] followed by anti-IL-12p40-Alexa Fluor 647 [Clone:C11.5 (BioLegend, Tokyo, Japan)] according to the protocol provided by BioLegend (http://www.biolegend.com/media_assets/support_protocol/Intracellular_Staining_Protocol_041515.pdf). Analyses were performed on a BD Accuri C6 cytometer (BD Bioscience). (TIF) [file pone.0129806.s002.tif]

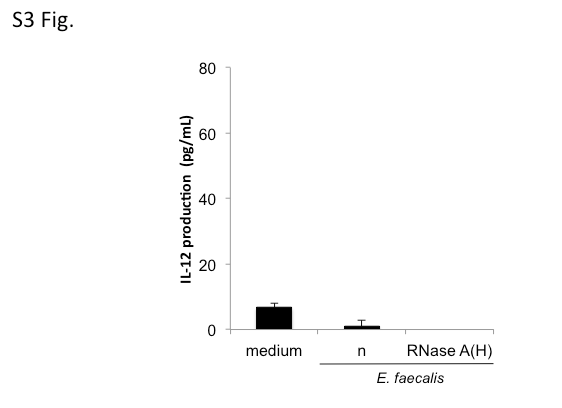

Supplement: S3 Fig — Heat-killed E. faecalis EC-12 was co-cultured for 24 h with CD14- cells sorted by a JSAN cell sorter (Bay Bioscience, Kobe, Japan). The IL-12 protein concentration in the culture supernatant was measured by enzyme-linked immunosorbent assay. mean ± SD, n = 4. (TIF) [file pone.0129806.s003.tif]
